# The Neurotic Origin of Disease, Etc.

**Published:** 1875-06

**Authors:** Frederic D. Lente

**Affiliations:** Member of the Council of the New York Neurological Society


### Selections.

(From the Psychological and Medico-Legal Journal.)

### THE NEUROTIC ORIGIN OF DISEASE, AND THE ACTION OF REMEDIES ON THE NERVOUS SYSTEM.

By FREDERIC D. LENTE, M.D.,

MEMBER OF THE COUNCIL OF THE NEW YORK NEUROLOGICAL SOCIETY.

Read before the New York Neurological Society, Dec. 7, 1874.

*Mr. President and Gentlemen :*

Moritz Romberg, Professor of Medicine in the University of Berlin, commences the preface to the first edition of his classical work on "Diseases of the Nervous System" with the following quotation : "I fear it will be a long time before combined efforts will enable a medical author to arrange and accurately describe the diseases of the nervous system. The position we at present occupy is a very inferior one. Such was the opinion of Sir Charles Bell. A decennium has since passed. Many laborers have been at work. We have a rich treasury of materials, and yet we possess no work on nervous diseases which meets the requirements of science. The blame lies, in a measure, with the distinguished members of our profession who have been deterred by a fear that pathological investigations would fail to cope with the advanced state of physiological inquiry; in others, the fault is to be attributed to that mental indolence which gives the preference to the easy path of tradition, and with foolish skepticism rejects everything that is new. But in no department of pathology has physiology exerted so great an influence, nowhere has free research achieved so glorious a victory over dull, traditional routine, as in the doctrine of nervous diseases."

This was in 1840. Let us see what he says ten years later, for he thus indicates the history of our progress in this direction: "The hope expressed in a former edition has only been partly fulfilled. The majority of students have been attracted by the school which seeks to base the science of medicine exclusively upon pathological anatomy and chemistry. This has given rise to new errors, as the doctrine of the crases most clearly shows. The study of nervous diseases, which some persons have refused to acknowledge as anything but the manifestation of other morbid processes, has been declared a fruitless research, and in some schools has been almost interdicted. If this was the case in the universities, matters were necessarily in a worse condition in daily life. Practitioners chased an illusion and caricature called spinal irritation, with which they satisfied their craving for explanation, and condensed neuro-pathology into a space which could be covered with the tip of the finger. To guard against greater debasement, we must enter again upon the path which the master-mind of Charles Bell, the Harvey of our century, has opened to us."

If this was the case in critical and classical Germany, how much more true was it in the case of other countries. A quarter of a century has rolled by since Romberg thus rather sorrowfully expressed himself. Where are we now? We are certainly not where the wonderful improvements in every other branch of medical science should have placed us. But the past few years have afforded indications of a brighter future. Minds of the highest calibre have been devoting themselves to this study. Monographs, treatises, and periodicals, in different languages and of great value, have appeared, to entice us on, and to light us along this difficult but most interesting path of scientific investigation; none more interesting, none more practically useful, in the estimation of those most competent to judge, both in this country and in Europe, than that of our countryman, Prof. Hammond. But the teaching of our schools, and our systematic treatises on therapeutics, and our hospital clinics, are far behind our special literature. Their pathological views, as a general rule, show but little change. Mr. Wallace Wood, in a well-written article in the last number of the Psychological Journal, indulges in the hope that "the next five years will inaugurate a new system, which will promise as much or more than the brilliant but premature scheme of Gall." I think we may safely predict a future like this for the therapeutics as well as for the physiology of the nervous system.

For some years past my own experience has impressed me with the belief that in most diseases, especially those of an acute character, the morbid impression is first made on the nervous system, either *directly*, as in the case of the mucous membranes, where the peripheral nerves are more accessible to the immediate action of toxic agents; or *indirectly*, through the medium of the blood. That the remedies, therefore, most likely to exert an *abortive* effect on disease; or, when that is not attainable, the speediest and happiest relief, are those which are supposed to act especially on the nervous system; and that our ordinary treatment, by acting in a more indirect, and, therefore, less speedy manner, as well as by disturbing other portions and other organs of the body not involved in the morbid process, is slower, less certain, and more unpleasant in producing their curative effects. I ask your attention, in the first place, to a review of the history and therapeutics of certain diseases not commonly considered neuroses.

And, first, of "malarious" fever, and especially the intermittent form, as this is a disease of paramount importance to very many of us just now; its unusual prevalence everywhere, but especially in localities commonly considered remarkably exempt, being one of the most remarkable of the inexplicable phenomena characterizing its history in all ages.

I have no theory as to the causation of "malaria" to support. I am as much confounded by its vagaries and seeming inconsistencies as any of its hosts of distinguished investigators. But whatever the cause may be, whether vegeto-aerial or diurnal vicissitudes of temperature and moisture, or both combined, or gaseous products, or the fungus theory, so ingeniously and learnedly set forth by the late J. K. Mitchell, of Philadelphia, it seems to act primarily on the nervous system, and its morbid energy to be most expended in assaults on that system.* The evidence adduced, both from the arguments and data of those adopting a certain theory of causation, and of those virulently opposing it, alike tends to support this idea. Thus it is admitted, I think, by all, that *repose*, and especially repose *at night*, renders the body most liable to miasmatic influences; while exercise, even in what is supposed to be the hotbed of the poison, is preventive. Every one knows how much more liable we are to "take cold" when sleeping, or even when in a drowsy state, and especially at night, unless the body has an additional covering, and how great a protection exercise is. In both cases the nervous system is more impressible in the one state than in the other. Exercise and occupation, in all *epidemics* of whatever kind, are the great preventives; indolence and fear, causing greater nervous impressibility, are our worst enemies. All army surgeons of large experience can corroborate this.

The sudden onset of the disease, instances of which abound in all their writings, point to the primary affection of the nervous system. "Exposure for a single hour at night," says Mitchell,† sometimes produces an almost immediate attack, sometimes causes a *tendency*, not expressed perhaps for some months." Mungo Park, alluding to African experience, says: "The rain had not continued three minutes before many of the soldiers were affected with vomiting, others fell asleep, and seemed as if intoxicated. *Twelve of the soldiers were ill the next day.*" He felt the same symptoms himself. The following cases, occurring lately at Whitworth Hospital, reported in the Irish Hosp. Gaz. (Boston Med. and Surg. Jour., Nov., 1874), afford an additional illustration: Two hospital nurses, accustomed to hospital sights, odors and sounds, were subjected to a most remarkably fetid odor from the alvine dejection of a dead body. Within ten minutes one fell down, vomited, and became unconscious. After several remedies, an ice-bag to the nape restored

* Virchow has lately said: "Fever consists essentially in elevation of temperature, which must arise in increased tissue-change, and have its immediate cause in *alterations of the nervous system.*"

† "Essays," edited by S. Weir Mitchell.

consciousness in a short time. The other nurse vomited while at the dead woman's bedside, went to her room, and fell down in an unconscious state. Half an hour after the ice-bag was applied to the spine she regained consciousness. Dr. McDowell was of opinion that the medulla oblongata was under the toxic influence of the noxious gases.

As explanatory of the manner in which the fever-poison may thus suddenly influence the system, I adduce a brief account of a very interesting experiment performed a few days ago by Dr. Horatio Wood, of Philadelphia, and of which he was kind enough to give me the particulars: He divided the cord of a dog at the junction of the *medulla* with the *pons varolii*, and, of course, above the vaso-motor centres. Although the lateral sinus was accidentally divided, an accident difficult to avoid in so high a division, and "three-fourths of a pint of blood lost," which should have *reduced* the temperature several degrees, there was a rapid rise to 104° F. (the normal temperature of the animal being about 102°). Here all the controlling influence on the peripheral nerves was lost, just as it is when a powerful toxic impression is made from without. *Cholera Asiatica* not unfrequently produces this sudden overwhelming effect on the nerve-centres, and patients drop suddenly.

Periodicity, or tendency to relapse at certain intervals, the most marked, by many considered the pathognomonic symptom of malaria, also indicates the nervous system as the seat of the disease. This and incubation, with which it is intimately connected, present, perhaps, the most interesting, the most extraordinary and inexplicable phenomena connected with the history of disease. That an exposure should not manifest its effects on the system for a week, or a month, or even many months, and should then, apparently without provocation, induce suddenly the most striking and even alarming symptoms: this phenomenon, this tendency to relapse, is often as annoying and perplexing to the physician as to the patient, and it is practically of the utmost importance that its mystery should be unraveled. The very accuracy as to time points to the nervous system as that portion of the body to which we should direct our investigations; since, if we look *within* the body for the cause, as the slow accumulation or the growth of the *materies morbi* to the point of saturation and explosion, the blood itself, or any organ except the nerves, ought, it would seem, to give some indication of diseased action. And, on the other hand, if we look *without*, or for external impressions, "exciting causes," the very suddenness of the attack would seem to indicate that it must be through the medium of the same system.* Many cases might be cited in which none but *nervous* manifestations follow exposure to malarious influence—neuralgia, especially supra-orbital, for instance. A man, surrounded by malarious disease in all its forms, recently applied to me with a most atrocious pain over the left eye, coming on every other day at or near a particular hour. He could not credit my assertion that it was "fever and ague," as he had experienced none of the ordinary symptoms whatever. But a few full doses of quinine sent him to his work rejoicing. Periodicity, as has been already remarked, instead of indicating malarious fever, is rather a characteristic of nervous disease. Aitken adduces the periodicity of the phenomena of fever as evidence, not that the fever is of a particular *type*, but that the *nervous system* is influenced; also, the benficial effect of quinine as corroborative evidence. We observe it markedly in epilepsy. Thus, in a case now under my charge, the paroxysms came on regularly every two weeks, on Thursday, even when the subject was at work, and feeling as well as usual. In another case the attack occurred every four weeks, at night, for a long period. In a third case, under the care of Prof. Hammond, which I saw to-day, and the particulars of which I obtained from the patient's mother, the fits, while the patient was under the influence of the amyl nitrite, taken three times a day, came on as follows: Four days' interval, 3 fits; 4 days' interval, 1 fit; 4½ days, 2 fits; 4½ days, 2 fits; 4 days, 1 fit; 2½ days, 1 fit.

The symptoms following the ingestion of poisons sometimes show marked periodicity. Christison, as quoted by J. K. Mitchell, informs us "that a whole family, consisting of a woman and four children, were attacked by a tertian fever by living for four months on *edible mushrooms*." The husband, who lived on other fare, escaped the symptoms. "Westerhoff observed, in those who

* There is sometimes a marked periodicity in *chorea*, and Dr. Weir Mitchell, who is eliciting some curious and interesting phenomena in his investigations concerning this disease, finds that the attacks *usually* recur in the spring.

were poisoned by mouldy food, an *intermittent somnolency*. M. Gassand saw cases of *ergotism*, where the sensations, either of heat or cold, were *intermittent*." Other writers mention this feature. "The *mental disturbance* intermitted in one case, *inflamed eyes* in another, and all the phenomena in a third." "The Dic. des Sci. Med. reports cases of this kind, in which occurred the *most acute pains* which were intermittent." In these cases it was also noted that the preparations of *bark* were the most effectual remedies. Dr. Mitchell adduces these facts, among hosts of others, in support of his fungus theory of "malaria." Whether he is right or not, they strongly support the position I am endeavoring to sustain. His essay will well repay perusal. But the most striking instance of marked periodicity occurring from a purely nervous disturbance, happened in a case published by me in the Amer. Jour. Med. Sci. for July, 1862. A girl, eleven years of age, and in fair health, not having been subjected to any malarious influence whatever, but of an exceedingly nervous temperament, received a wound of the supra-orbital region, from a fragment of copper-cap, producing amaurosis. The removal of the foreign body almost immediately relieved the defect of vision; but, on the second night after, the eye became much inflamed, deep-seated pain set in, nausea, coldness, and "numbness" of the extremities, and great nervous excitement. Pulse and skin natural; no fever. Anodynes were rejected by the stomach, and the pain was much mitigated by tinct. aconite locally applied. Next morning was pretty comfortable. This was on the 8th of July. On the 10th, another severe attack supervened; the pain was particularly severe in the back of the neck, with inability to flex the neck forward. Skin hot, pulse frequent; vomited. Ordered mild antispasmodics. Fell asleep after one dose, and slept quietly. In the morning quite comfortable; skin pleasant; pulse natural. On the 12th, had a similar attack, except that the skin and pulse were but slightly affected. One dose put her to sleep. She was now ordered a grain of quinine, as a tonic, three times a day. Her next attack, on the 14th, was similar but very slight. She had been ordered five grains quinine on that day as a prophylactic. On the 16th had a violent attack of pain in the eye, but no other symptom. Quinine had been omitted by mistake. The eye looks much worse, and vision is much impaired. July 30th:

Vision perfect. Dec. 14th: Patient has continued well, but has *visus dimidiatus* of the affected eye.

Robert Jackson, who wrote on this subject three-quarters of a century ago, seems to have had a clearer idea of the causes and effects of malaria than any writer of more modern times; and had his knowledge of the anatomy and physiology of the nervous system been equal to that of the present day, might have solved some of the intricate problems which he have perplexed us ever since. All his theories, his experiences, and his observations seem to converge towards the nervous system as the *fons et origo* of the diseased action, and to the necessity of sustaining this, and protecting it from morbid influences, not by any special remedy, but by a combination of judicious management, hygienic and therapeutical, moral and mental, as well as physical. Were it not for fear of rendering myself tedious, I would like to quote a page or two from his work, modestly entitled, "Remarks on the Constitution of the Medical Department of the British Army," published in 1803, which may be found in the library of the New York Hospital.

It may seem to be diverging from our line of investigation to cite *articular rheumatism* as an example of a neurosis. But I ask your attention, for a few minutes, to some well-known facts in its history and treatment, and also to a remarkable memoir of Dr. J. K. Mitchell, to be found in the volume of essays to which allusion has already been made. He attempts to prove rheumatism a neurosis, and refers its phenomena all to a spinal origin. His cases show a striking resemblance, in their articular symptoms, to acute rheumatism; and that these are caused by the spinal disease or injury, he fully demonstrates. But they all seem to be cases of organic disease of the spine, or injury of the *medulla*, except one, which I abridge.

Robert Gordon, well known as the carrier of Poulson's Daily Advertiser, 56 years of age, vigorous constitution and active habits, was the subject of the following attack. Observing a severe pain in his right heel and ankle, immediately followed by redness, heat, and tumefaction, he caused himself to be largely bled, and took salts and magnesia. On the following day the symptoms increased and the ankle and knee of the opposite side becoming affected, he was confined to bed.

On the third day, my first visit was made. Had then a full, strong, frequent pulse, flushed face, dry skin, whitened tongue, and complained much of the severity of the pain in his legs, and his incapacity to endure the slightest pressure or motion. I directed the application of seventeen cups to the lumbar region, so as to abstract twelve or sixteen ounces of blood. Next morning, I found the pain almost entirely gone; does not complain of moderate pressure, and is able to move his legs without inconvenience. Ordered a draught of salts and magnesia, and a lotion. Third day: Pain in legs scarcely perceptible, but the shoulders, elbows and wrists are beginning to exhibit marks of severe inflammation expressed by pain, tumefaction, heat, and redness. Ordered twelve cups to the cervical spine. Fourth day: Patient sits up; complains of stiffness, but no pain except in one wrist, and that very slight. Epsom salts and magnesia. Fifth day: Finding nothing for which to prescribe, arranged patient's diet, recommended occasional use of aperients, and took leave of the case.

Called on tenth day to inquire into the results, and found there had been no return of the disease. Since that time a severe winter has passed, and he has continued in a good state of health.

The reader will perceive, says the reporter, that the general bleeding, though very copious, proved of no service, and that the large local bleeding in the lumbar region benefited solely that part of the disease which lay at the peripheral extremities of the nerves supplied by the lower end of the spinal marrow. The inflammation in the upper extremities continued afterwards in progress, and was arrested only when cups were applied over the cervical spine. In this connection I refer you to an article on "Neuroses of the Joints," in the Sept. No. of The Psych. and Med.-Leg. Jour., translated from the German of Moritz Meyer. In concluding his essay on rheumatism, Dr. Mitchell adverts to "the close connection between rheumatism and certain diseases of the mucous and fibrous tissues of the eyes, nose, mouth, alimentary canal, bladder, and urethra. In many cases, diarrhœa and dysentery are found to alternate with rheumatism of the extremities, and particularly of the lower limbs; wherever such cases happen, they are always found to yield more readily to spinal treatment than to any other mode of cure, thus affording another proof of the spinal origin of these cases." In a case of severe pruritus, reported in the first number of the Archives of Dermatology, by Dr. Beard, for Dr. Kinsman of Ohio, the latter remarks: "The moment the current (of electricity) is passed along the spine, the pruritus ceases." Dr. Beard also reports a case of herpes zoster frontalis for Dr. Bulkley, cured by galvanism. I have thus also promptly relieved cases of herpes zoster. The spinal origin of many cutaneous affections is now well established, therefore such illustrations need not to be multiplied. With reference, however, to the last named disease, I desire to refer briefly to some remarkable evidences of its purely neurotic character, as well as of other veiscular affections, contained in M. Vulpian's recent French edit. of S. Weir Mitchell's "Lesions of the Nerves," and hence, to the very great importance, in all cases of very obstinate eczematous or bullar diseases, of paying special attention to the influence of the nerve centres, and of possible nerve lesions, as a cause. One case is that in which a ball, traversing the chest, without injury to the skin, except at points of entrance and exit, induced a very painful herpetic eruption. Brown-Sequard mentions a contusion of the brachial nerve causing a herpetic eruption all along its course. Sometimes the eruption is analogous to that of pemphigus. M. Raynaud cites a case in which an injury of the trunk of the "cubital nerve" was followed by an eruption along the whole course of the nerve. "The list of such facts," says Mitchell, "may be extended as far as one may wish." He says that his term "eczematous" to such affections has been sharply criticized by Charcot and Handfield Jones. How little should we regard the trifling eruption, sometimes only lasting a few days, which marks the onset of *herpes zoster*, when the neuralgic symptoms are so much more pronounced and so difficult of cure; sometimes, indeed, under the old methods of treatment, lasting through the remainder of life. Electricity now holds out a prospect of a speedy cure. In fact, this disease is as much out of place in a work on *skin* diseases, as a whale would be in a treatise on fishes.

The very great uncertainty attending the classical treatment of rheumatism through the blood, and the effects of other treatment acting in a totally different manner, do not give much support to the theory of blood-poison. Quinine I have found to act sometimes with great promptitude in aborting the disease when acute. even when there is no evidence of malarious poison, Some rely upon it as a routine treatment. M. See, of La Charite, remarks in a lecture recently delivered there, that he considers it of great value in acute rheumatism, and always returns to it with benefit after a trial of all other remedies. He says, "Its effect is on the spinal cord, in lowering its irritability, and thus diminishing the sensibility to pain, and lowering reflex excitability, thus reducing the afflux of blood to the inflamed joints." "It may be mentioned," says the Lancet, August 8, 1874, "that this mode of treatment is adopted by a large number of the leading physicians of Paris, either alone or with other means, and they all appear to be unanimous in its favor." But to be successful, the drug must be administered with no niggard hand. The blister, or counter-irritant treatment as it is sometimes called, is not seldom promptly successful, if carried out with the firmness which only will insure success. The blisters require to be large, and to be applied freely, and rarely give any annoyance. Patients often beg for their repetition. Ice to the joints is a favorite and successful remedy with some. Esmarch and others used it with great success in the Franco-German war. Dr. Da Costa has recently used the bromides successfully.

The application of cold to the whole surface of the body, or cold bathing, packing or affusion, in cases of extreme danger both in rheumatism and *fevers*, is a still more potent method of treatment, and affords, perhaps, a still stronger evidence of the neurotic character of these diseases. When the temperature in these cases runs up to 109° or 110°, though it may go still higher, it was found that death was the almost inevitable result, until after the adoption of a certain bold treatment, which consists in a rapid reduction of the abnormal heat by the application of cold to the whole surface.* It appears to be generally understood that the good effect is due to the direct abstraction of heat. But it is doubtful if this has much to do with it, if it has anything at all. Frequently the internal temperature continues to fall for some time after the patient has been put into bed and covered up, to such an extent as to threaten serious consequences at times. Now, the mere abstraction of heat by direct action of the cold must cease the moment the cold is withdrawn from the surface, and we must look for some explanation of the continued fall of temperature. Then there are cases in the so-called hysterical

* The treatment is not new as regards fevers, but was proposed and largely practiced by Currie, but fell into comparative disuse.

conditions of females, where the surface is habitually cold and clammy, and yet the best remedy is the cold shower bath. I am happy to be able to adduce the experience of so able and careful an observer as Robert Jackson, who speaks, as regards fevers, from an experience of the largest kind. "It is possible," he says, (opus cit.), "that excess of heat may exist, and it actually does exist, without *superficial excitability*, that is, without a due share of sensibility of surface (italics mine) both in the early period and in the later stages of fever. Such condition of fever is common in spring, common in Europeans soon after their arrival in tropical climates, both in the commencement and in the after-period of the disease, either as connected with plethora, or with internal congestion. The heat is then often ardent—particularly on the trunk of the body. A thermometer, in this case, is a fallacious guide. It indicates a high temperature, but experience proves that cold bathing does no good; it probably does harm in the cases connected with internal congestion. To trials, in such cases, it is believed the credit of the remedy has been sacrificed in the fever of the West Indies, and probably in that of America." That is to say, in modern phraseology, the cutaneous nerves must be in such a state as to receive and convey an impression to the nervous centres, thence to be transmitted to the vaso-motor nerves of the internal organs, contracting their minute vessels, diminishing hyperæmia, arresting, for the time at least, destructive metamorphosis of tissue, and over-production of animal heat.*

As a remarkable illustration of the powerful and sometimes destructive effect of applications to the surface, when too extensive, too severe, or too prolonged, I remind you of the results which followed the old experiment of varnishing animals, and the death of the child coated with gold leaf, to represent the golden age, in a procession during the reign of Louis XIV. The cause of death in these cases has been variously attributed to the great fall of temperature which ensues, to the complete arrest of perspiration, to asphyxia, etc. But lately, Dr. Feinberg has been reinvestigating the subject by

* It is not contended that this is the only mode by which the impression may act on the nervous centres. In fact, the *inhibitory* action developed in those centres, as will be shown by examples, may be still more powerful, even to the production of fatal consequences.

further experiments, and has come to the conclusion that it is "from paralysis of the *vaso-motor nerves* all over the body, producing excessive dilatation of the vessels, and extensive extravasations. In the spinal cord, especially, was there congestion and extravasation." There was decided sinking of the temperature. He attributes the vaso-motor effect to intense irritation of the sensory nerves of the skin. His specimens were examined by the celebrated histologist, Frey, and his facts confirmed by him.

The coincidence of *excessive dilatation* of the capillaries of the viscera, with marked *reduction* of temperature, may seem to conflict with the views frequently expressed or implied in this paper, as to the increase or diminution of temperature from vasal dilatation or contraction respectively, though we have been careful not to attribute it exclusively to this. But we must not forget that, in Feinberg's experiments, this vaso-motor paralysis is carried to an *extreme* degree. In like manner, though the physiological effect of some of the drugs, to which your attention will presently be asked, is to *contract* the vessels, they are found, if pushed to an extreme or fatal degree, to do just the opposite; and this ought always to be borne in mind in estimating the peculiar properties and therapeutic value of these powerful agents. So here, the immense reduction of the intra-vascular pressure, producing sinking of the heart's action, and almost complete arrest of the circulation in the capillaries, would naturally induce a fall of temperature; whereas the varnishing of a smaller portion of the surface would have doubtless produced quite a different condition of the visceral vessels.

To return to a consideration of the *joint* affections of rheumatism, I desire to remind you of the many instances where pain and inflammation of the larger joints supervene on injuries, and suppurative diseases of the extremities, and on other conditions giving rise to what is usually called septicæmia or pyæmia, supposed to be due to blood-poisoning, or to a low grade of inflammation extending along the veins and absorbents. A patient of mine, a lady eighty-three years old, recently died of senile gangrene of the right foot, advancing slowly at first, but finally running up the leg with great rapidity, attended by no chill or febrile reaction, but affecting the sensorium profoundly; simultaneously was developed a "rheumatic" inflammation of both wrists, and for some days the only pain which she appeared to suffer was due to this. Here there was no evidence whatever of blood-poisoning. In view of this, and many similar facts, it would be well to review our ideas of the causation of these intercurrent phenomena, and to remember the connection which may exist between what has been said about neuralgia and the spinal origin of rheumatism, and the fact that in the cases just mentioned, those of supposed septicæmia, quinine is now considered the sheet-anchor. I am told that Sir William Gull, twenty years ago, claimed a spinal origin for rheumatism, and thought the idea original, but was preceded a quarter of a century by our distinguished countryman.

We are, of course, not able to experiment on the human body as we do on animals, in order to solve physiological problems, but accident frequently comes to our aid; and the evils of war are often turned to good account. I desire to call your attention to a few instances of this kind, which serve to elucidate the influence of the nervous system in the production and cure of disease.

The following case fell under my notice while on a visit to Saratoga Springs, in the autumn of this year, and, I think, demonstrates that which had only been previously done by experiments on animals, what Austin Flint, in his "Physiology," calls the starting-point of our definite knowledge of the functions of the sympathetic nervous system—"that the influence of the sympathetic nerve in the neck was propagated from below upward toward the head, and not from the brain downward." This was carried further by Brown-Sequard in 1852, whose experiments led to the discovery of the vaso-motor nerves.

*Case.*—Theophilus S., stair builder, fell, on June 22d, 1874, twenty-five feet; the spinal column, at the base of the neck, and from that to the right shoulder, striking a hand-rail. The blow was thus received rather on the right side of the spine than directly upon it. Had always been well, and had never been injured until the Franco-German war, when he was stunned while in charge of an ambulance by the explosion of a shell. He soon recovered, and is not aware that any ill consequences followed. Last winter he was greatly chilled by a somewhat prolonged exposure, since which he has not been quite so well as usual. After his fall, he was unconscious for an hour. Dr. Whiting saw him at once. There was no indication of any injury to the head. On returning to consciousness he complained of a most intense pain in the occipital region, low down ; the least movement of his body gave him intense pain at *seat of injury*, and nowhere else. He also felt as if the whole right side, the arm, leg, etc., "were dead." No uneasy sensation in left side. His vision was also impaired, and he had "noises in his ears." He required a hypodermic dose of morphine for his headache. Was constipated for several days, and had retention of urine, lasting three days, and then passed his water, and continued to do so. For five days could not move his right lower extremity at all. Could move his fingers, but any attempt at movement of the arm produced violent pain in the spine. Motion and sensation in the limbs returned very gradually. For a while the headache required anodynes, and gradually subsided.

Five weeks after the accident he felt so much better, that, with the aid of a stick and limping, he walked about a quarter of a mile to the doctor's office. A day or two after, he was much worse, and was put to bed again. Severe gastric and abdominal symptoms set in. After eating one or two mouthfuls of food, there was so sudden and enormous an accumulation of gas that he felt as if he would burst ; had other symptoms of indigestion, and an obstinate diarrhœa set in. Headache also returned, and required the hypodermic injections, which acted very slowly. About eight weeks after the first relapse he felt so much improved that he went to work ; worked only two hours and a half, and with great difficulty. Pain commenced in his shoulder and back, then his fingers stiffened so that he could not hold his hammer ; headache set in, and his old diarrhœa ; he could scarcely get home on account of the latter, stopping three times to relieve himself. He was better, after a rest in bed for some days. It was soon after this, when he was able to walk about a little, that he came under my notice in Dr. Whiting's office. His prominent symptoms were as follows : vision imperfect ; since his first relapse sees everything sometimes blue, sometimes red ; and, on lying down, sees things "quivering," and in this position his headache, now very moderate, is decidedly increased ; sleeps badly, was somewhat relieved by moderate doses of bromide of potassium. Sleeps pretty well when *sitting* in an easy-chair, and better in the daytime. His diarrhœa is uncontrollable by opiates and astringents, except for a short time. When his bowels are loose, his micturition is painful. The *sudden* puffing up of his stomach, on taking a few morsels of food, was a marked and intractable symptom for some time, but he is now, in a great measure, relieved. Another very curious symptom has lately appeared; his evacuations are attended by an intense burning sensation in the bowels and around the anus, "*as if sprinkled with red pepper.*"

I have not the space to comment on this remarkable case. I showed him to Dr. Hammond, and Dr. Cross also saw him. They could not make up their minds that all his cerebral symptoms were due to the spinal injury. Perhaps they were not. But the abdominal symptoms certainly were; and, in connection with this, and with the remarks on the spinal origin of rheumatism, and the nervous origin of fever, I adduce other cases of abdominal disease caused by and causing affections of the nervous system, and relieved by remedies addressed to that system.*

A remarkable case of convulsions and coma, caused by a slight abdominal irritation, is related in a letter from Paris, from the parent of the patient, and published in the Boston Med. and Surg. Jour., for Oct. 29, 1874: "The child while at dinner, and in perfect health, was suddenly seized with a convulsion of a most violent and alarming nature, becoming rigid, eyes fixed, pupils much dilated, teeth clenched, accompanied by the most distressing gasping for breath, and complete unconsciousness. The physicians summoned tried the warm bath, artificial respiration, etc.; the *hot* bath, dashing cold water. One proposed a hypodermic injection of morphia, which seemed to relieve the violence of the spasms. Sir John Rose Cormack arriving, suggested another hypodermic injection, and in case of failure, *tracheotomy.* The injection fortunately succeeded; he became narcotized, after having been in violent convulsions for two hours. The diagnosis was *worms.* The following day he awoke quite relieved. He subsequently passed two or three worms.†

* See case, very similar, in many respects, to that of Shires, in London Practitioner for March, 1874, extracted from Deutsch Archiv fur Klin. Med 1873, xi. The very first application of the galvanic current to the left cervical sympathetic relieved the violent cerebral symptoms materially.

† See a remarkably interesting case of most serious cerebral symptoms, caused by constipation, in Brit. Med. Jour., August 8, 1874; Med. News and Lib., Oct., 1874. Since the above account was writtten, I have met with a full and extremely interesting account of its history, in the London Medical Times and Gazette, from the pen of Sir John himself. This I propose to notice more fully in connection with a case treated by me within the past week.

I had a case very similar to this, in a child nine years old; the coma was complete for some hours, and death seemed impending. I also diagnosed correctly, as the event proved. There is really nothing remarkable about these cases, except as regards the age. Scores of *infants* in our large cities die every summer from convulsions induced by abdominal irritation and reflex action, many of whom might be saved by a better appreciation of neuropathology, but especially by a more liberal use of neurotics, and by bolder doses of *opium*, with the temporary aid afforded by chloroform inhalation. Reflex meningeal irritation is still constantly mistaken for inflammation, and the traditional horror of opium in meningeal and cerebral diseases of infants still stays the hand of the physician, and consigns many a curable patient to an early and untimely grave. In an article on the treatment of vomiting by electricity, published in the second number of Beard's "Archives of Neurology and Electrology," will be found three cases there related by me, remarkably illustrating the nervous, or, if you choose, malarious origin of diarrhœa, and its prompt arrest by electricity, after total failure of all other ordinary remedies—the cases of Mrs. L., Mrs. M. C., and the girl A. D. In the first, however, the extract of ergot, given for a different purpose in large doses, by hypodermic injection, was largely instrumental in the cure. And here I will supply an important omission in the case of Theôphilus S. I suggested to Dr. Whiting the use of ergot and bromide of potassium for the diarrhœa; and doses of half a drachm (fl. ex.) increased to a drachm of the former, with half-drachm doses of the latter, relieved him completely in one week. The fluid extract of *Eucalyptus Globulus* (which, however, differs from ergot in possessing astringent properties) has a similar effect to ergot in these cases, an effect not due to its astringent action, as proved by the previous failure of other more active remedies of this class. A combination of the two is excellent; and the powerful tonic property of the *eucalyptus* aids essentially. Prof. Hammond has found this combination admirable in a similar condition of the mucous membrane of the bladder, arising from an atonic condition of its muscles, as well as the muscular coats of its vessels, and constituting catarrh of the bladder, with an alkaline condition of the urine.

Dr. Austin Flint, Jr., in his recent work on the "Physiology of the Nervous System," publishes the results of the experiments of Dr. Moreau, in Paris, in

1860, which are strikingly illustrative of the preceding suggestions and cases. "In those experiments the abdomen was opened in a fasting animal, and three loops of intestine, from four to eight inches in length, were isolated by two ligatures. All of the nerves passing to the middle loop were divided, taking care to avoid the blood-vessels. The intestines were then replaced, and the wound in the abdomen closed with sutures. The next day the animal was killed. The two loops, with the nerves intact, were found empty, as is normal in fasting animals, and the mucous membrane was dry; but the loop, with the nerves divided, was found filled with a clear, alkaline liquid, colorless, and slightly opaline." This experiment has an important bearing on the pathology of *cholera Asiatica*, *the profuse watery diarrhœa, without abdominal pain*, indicating mere want of *innervation*, rather than an attempt to *eliminate a poison*, and the *subsequent* cramps in the voluntary muscles, pointing very decidedly to the nervous system as that mainly, if not exclusively, affected by the poison, and to the necessity for a treatment which may present, at least, more uniformity of action than any which has heretofore been devised. For the history of this destructive disease, as far as *treatment* is concerned, is far from creditable to us as a profession; those methods, based on totally different pathological views, showing about an equal amount of mortality.

The peculiar effect of our *cathartics*, especially when they are given for their so-called *derivative* action, can only be explained by their reflux influence on the brain, and its vaso-motor vessels, rather than by unloading the *abdominal vessels*, which is also a means, but a more tardy and less efficient, since the impression felt in the brain is almost instantaneous. This is felt sometimes even after an ordinary evacuation, or even after the passage of gas. In the same manner a sharp pain in the cardiac region is instantly relieved by the sudden passage of gas from one portion of the intestines to another, the pressure on certain nerves being thus removed. All these apparently trifling phenomena, unimportant individually, are valuable when studied together.

Returning to the consideration of accidents and the influence of nervous shock in the production and cure of nervous affections, I merely refer to the two following cases published by me while house-surgeon to the New York Hospital. A negro with delirium tremens, upon whom I had vainly exhausted all my remedies, broke from his attendant and sprang from the third-story window, falling on the stone stoop below, and striking on one buttock. He was carried to his ward, was found to have sustained no damage save a severe bruise at the seat of injury, and in a state of perfect sanity. He was discharged cured in a few days, when able to walk. At about the same time Dr. Lyman Stone, then house-physician of Bellevue Hospital, published a similar case. The patient was wildly delirious from typhus fever, and the case concluded with this quaint sentence: "He broke from the orderly, jumped out of the third-story window to the area below, and had a rapid recovery." A case somewhat similar was related to me a few days ago, by an intelligent lady patient. A friend of hers, sojourning at Litchfield, Connecticut, during the past spring and summer, accidentally fractured her forearm. She was of a nervous temperament, and the shock was very considerable. The case did well, but the point is this: For forty years regularly she had had a recurrence of "hay-asthma" at a particular date; but this summer has passed with no symptom of it. The case is particularly interesting in view of Dr. Beard's recent researches into the *nervous origin* of this disease, and the means suggested for its cure, of the efficacy of which he is very sanguine. See his paper recently read before the "Public Health Association." Now, I do not recommend or propose to employ such radical means of cure; but, in connection with the pathological questions involved in this essay, and the effects of the neurotics already alluded to, and those to be considered, they are suggestive.

In connection with the preceding reflections and hints —for I cannot call them arguments—obscure and uncertain though they may be at present, let us briefly review the physiological effects of some of the more important *remedies*, which, with the aid of a larger experience and a more extended series of experiments on living animals, may enable us to take advantage of what, it is hoped, may prove better views of pathology.

Among the more important of these presumed to act specially on the nervous system, may be included *atropia*, *ergot*, *strychnine*, *opium*, *conium*, *physostigma or eserine*, *iodine*, *calomel*, (in *sedative* doses), *quinine*, *phosphorus*, *amyl nitrite*, the *bromides*, the *anæsthetics* (by inhalation), *digitalis*, *electricity*. Many others might with propriety be added, but these have been most thoroughly studied as yet. *Guarana*, or Paullinia sorbilis, ought not to go unnoticed, though we know nothing of its precise physiological action; but the prompt relief which it affords in various headaches, including *migraine*, and the safety with which it may be administered in almost any dose, renders it, like the bromides, doubly valuable.

*Electricity* is placed last, not because it is the least in value, but because it is undergoing a thorough investigation, and its status is very unsettled. Judging from the power which it is rapidly developing over a great variety of diseases under the close study and thorough experimentation of many of the ablest men in the profession, it bids fair to stand high in the list of this class of remedies, and perhaps second to none in the Materia Medica. As an *analgesic*, and even *hypnotic*, it may, in a great variety of cases, aspire to a rivalry with opium—the *magnum donum Dei*—in some cases being far superior to it. If time will permit, I hope to adduce numerous cases in proof of this assertion, some of them very striking, a few of them I think *unique*, many of them not involving anything novel or strange to those of you who are in the daily habit of employing this agent in its several forms largely in your practice. But, as yet, comparatively few even of those considered the best therapeutists of the profession, make any considerable or systematic use of electricity, except in a very contracted range of diseases, and are extremely skeptical as to the reports from specialists, of its wonderful influence over symptoms and diseases to which it had heretofore been considered entirely inapplicable. It is no wonder that the extraordinary assertions of Remak, Duchenne, and lately Arthius, in Europe—to say nothing of their distinguished followers in our own country—should be received with discredit, and even with derision; it has invariably been the fate of all remarkable discoveries in our science. But an honest and careful imitation of their methods, uninfluenced by blinding prejudice, will soon convince the most skeptical that enthusiasm has but little colored or warped their reports; unless, indeed, the enthusiasm engendered by the almost magical results of his first essays should warp his own judgment. *Static* electricity has fallen into such complete disuse of late, and the little book lately published by Arthius is so peculiar and enthusiastic in its style, and so sneeringly alluded to by its reviewer in the last number of Hays' Journal, that I am induced to put on record here probably the first authenticated case of its curative effect in this country, and the first instance where it was used for *myalgia*, a disease for which we now know electricity is a specific. The operator was my colleague in the Council of the University of New York, the well-known divine, Mancius Hutton, D.D., who will, I hope, pardon me for using his name in this connection. He was experimenting fifty years ago with a cylinder machine made by himself, when his father happened to be suffering from an obstinate attack of myalgia of the muscles of the shoulder and back; he placed him on an insulated stool, also made for the occasion, charged him with electricity from his machine, and then drew sparks from all the affected parts. He was promptly and permanently relieved.

The physiological action of *ergot* is now pretty well established. It contracts the minute vessels, both arterial and venous. It also acts on the involuntary muscles themselves, as, for instance, the heart and uterus. It acts both on the vessels of the medulla spinalis and the brain and its membranes; this, and the fact of its being perfectly innocuous, even in very large doses, renders it, like the bromides, an extremely valuable medicine. The two together, and aided sometimes by belladonna, which has on the vessels a similar action, will probably prove one of our most reliable means of combating both acute and chronic affections of the encephalon. In spinal congestion, Prof. Hammond's very large experience has taught him that, when perseveringly used in large doses, it is a most valuable resource. Much has lately been written about its influence on fibrous tumors, especially of the uterus. I can confirm the reports of its great value in these cases. The tumors, if large, rapidly diminish, and their more serious complications, as hæmorrhage, are promptly relieved. Hypodermic injection is the most efficacious means. Many seem to have been deterred, after some trials, by the occurrence of inflammation, abscess, etc., and have tried suppositories of Squibb's solid extract, which succeeds, but not so well. Dr. Murdock and I have used the injections freely in one case (alluded to in my paper on vomiting, and on the hypodermic treatment of intermittent fever); 167 injections of Squibb's fluid extract were used, in many instances repeated in the same locality, after the resulting indurations had subsided, and only once with any untoward result. I attribute the effect on these fibroids to *starving them* out, by its constringing the vessels. This explains why it succeeds better on large tumors which are less nourished. When the body is poorly nourished, heterologous or adventitious growths are the first to be absorbed, and some of those tumors have been cured by the starvation system. Langenbeck thinks he has cured aneurisms of large arteries ; indeed German surgeons have published several cases of cure, but the experiment does not seem to have been followed up, and it seems difficult to account for its effect on such large sacs or their contents. Lately it has been injected in the cellular tissue, over large varices, with a promptly curative effect ; here its action is easily comprehended.

Drasche, of Vienna, has lately been credited with being the first to recommend and to use hypodermic injections of ergot for hæmoptysis and other hæmorrhages. But in October, 1869 (see report in Medical Record for November), and before the publication here of Langenbeck's trials, I used the undiluted fluid extract of ergot in a desperate case of post-partum hæmorrhage (20 to 30 minims), and the patient recovered. Its beneficial effects on certain forms of diarrhœa have already been alluded to, and its successful employment by one German and several French physicians in severe diarrhœa and dysentery, is alluded to by Wood in his recently-published treatise (op. cit.). It is well to combine it with *eucalyptus*, which, there is good reason to believe, acts similarly on the capillaries.

Dr. Crichton Browne, in the London Practitioner, for June, 1871, and E. Churchill Fox, of the West Riding Asylum, in West Riding Asylum Reports for 1871, give clinical evidence of the excellent effects of ergot in mania. The latter makes the following important statement: "It has sometimes occurred to me that the bromide of potassium, the value of which I would be far from depreciating, has a tendency in some cases to aggravate the attacks of epileptic mania. It seems to relieve the muscular at the expense of the mental element in the epileptic condition. The fits are reduced in number and severity, but the paroxysms of mental disturbance are intensified and prolonged. Under such circumstances ergot becomes of the highest service ; its use, alternated with that of the bromide of potassium, places the two phases of the malady under equal and powerful control." I have only space to say a word about the *dose* of this valuable drug.

If it is used timidly or sparingly, or with a dread of the horrible consequences of *ergotism*, which has been too long the traditional raw-head and bloody-bones of routine practitioners and authors, it would be better to try some other drug. Less than *one drachm* of a good fluid extract (ergotine, so called, has no existence), three times a day, for an adult, ought to be the minimum dose, though we may *commence* with half a drachm merely out of respect for the stomach. In some cases of a very dangerous or obstinate character, the dose may be larger; and if for acute disease, or if not to be continued very long, very much larger. "In one of Wright's experiments," says Wood, "an amount equal to two drachms for every pound weight of the dog, failed to kill." Horatio Wood says, "Although I have given ergot (fluid ext.) in ounce doses, I have never seen it cause any distinct symptoms." He alludes, however, to two cases, in which poisoning did occur; but the amount taken was not mentioned by the reporters. I cannot explain the statements of the numerous writers, who, for hundreds of years, have given accounts of fatal epidemics of ergotism, except by referring you to the testimony of Trousseau and Pidoux,* who "assert that these epidemics are not dependent on any specific action of ergot, but are either epidemics of blood diseases, or simply the results of improper and insufficient food—merely the outcomes of poverty, wretchedness, and famine." I have not referred to all the diseases for which this remedy has lately been successfully tried, but have devoted to it more space than I should have done, had not my friend Dr. J. C. Peters passed it over in his valuable paper of last month, on the vegetable neurotics.

I ask your attention next to *digitalis*, both because of its great value, and because it affords another still more striking instance of the evil of traditional evidence, or rather of a blind reliance on it. Up to a quite recent period in the history of medicine it was looked upon as, *par excellence*, a depressing remedy, especially on the circulation; and I imagine that but comparatively few physicians now, taking the whole country, have any definite idea of the value of this remedy as a *heart tonic*, and of the great range of its applicability in disease. The error has arisen, probably, as errors concerning the action of remedies have too often arisen, from noting the effects of *toxic* doses on animals. In *such doses* "it lowers

* Horatio Wood's Mat. Med. and Therapeutics, p. 469.

reflex activity, and induces lassitude, prostration, muscular tremblings, and sometimes convulsions." Its powerfully *tonic action* on the heart has been demonstrated by all the numerous experimenters on its action on animals. It produces such an energetic action on the heart, if pushed too far, "that the ventricles become white, as the last drop of blood is squeezed out of them." Finally, if pushed to its poisonous effect, the heart is arrested in systole (Wood). In the case of a lady at West Point, a patient of Dr. Peaslee's, who had had a peculiar affection of the heart for some years, the pulsations being above a hundred, and very feeble, becoming extremely rapid, feeble, and irregular on walking across the floor, and who had been told, as she said, that there was no remedy for it, I prescribed tincture of digitalis in only ten-minim doses three times a day; within forty-eight hours, if my memory serves me, the pulsations of the heart and the throbbing of the vessels of the head and neck became alarming, so that in my absence she sent for Prof. Dunster, then the assistant surgeon of the post. He stopped the remedy, finding her pulse regular, full, and strong, and not abnormal in frequency. This was the end of her heart-trouble, for, I think, a year, when a similar attack, but much milder, was relieved by her physician at home, to whom she related the effect of the remedy in her previous attack. This is a typical, but not an uncommon case. It is not only in such marked cardiac cases that digitalis exhibits its great value as a neurotic. In all cases of debility requiring vegetable or mineral tonics, and when the heart's action is disproportionately weak, as is frequently the case, I employ it as an auxiliary, and with prompt and decided effect; also in combination with these remedies, or with astringents in passive hæmorrhages, especially in some obstinate menorrhagias. It has been shown, by direct experiments on frogs and rabbits, that it rigorously contracts the capillaries like ergot and belladonna (Boldt and Ackermann, quoted by H. Wood). Where an indication for the remedy seems to exist *from the weak or irregular action* of the heart, and a *concentric* hypertrophy of the latter is made out or suspected, the latter being usually considered a strong contraindication, it is well to give it, but to do so cautiously; and as the remedy is pretty uniform, and in my experience, and contrary to tradition, also *non-cumulative* in its action, it is perfectly safe; and I have seen it very efficacious in those very cases. In sudden cases of prostration from disease, or poisoning by such agents as paralyze the heart, as aconite or veratria, the remedy may be given by hypodermic injection. In Bellevue Hospital I am told it has been used thus with prompt effect in sudden and dangerous suppression of urine, though its action on the kidneys is probably secondary to that on the heart and arteries, and not as a *diuretic.* It is well worth trying, commencing with ten minims. In severe cases of cardiac irregularity and frequent pulse, when the patient is not confined to the recumbent posture by necessity, it is better to enjoin it while the remedy is being tested, as it not only produces its effect by *direct action* on the muscular fibre, increasing its power, but by an inhibitory action on its nerves, allaying irritability and irregularity. It passes my comprehension how so advanced a man as Prof. See, of La Charite, can declare that digitalis is a cardiac depressor or paralyzer, and that Liebermeister and Headland should substantially sustain this opinion. This drug will be again alluded to under the head of paralysis of the heart.

Few, it is presumed, will be willing to admit *calomel* into the list of neurotics, or of remedies acting directly on the nervous system, and I can only refer you to actual clinical experience in my own practice and in that of others which seems to me to place it beyond doubt. I have not time to give recent cases, but I refer to numbers already published by me in the New York Journal of Medicine, March, 1856, in an article on Dysentery, and in the same journal for March, 1870, in a paper read before the Dutchess County Medical Society on the "Sedative Action of Calomel." As this peculiar and important action of calomel is scarcely at all comprehended at the present day, not even alluded to by Waring, Phillips, and Sidney Ringer, in their late editions, though well known to James Johnson, Annesley, and many other distinguished topical writers of their period, as well as to Hamilton of Edinburgh; as it is entirely descredited by Headland, and not even referred to by H. Wood in his able and very valuable, though, in some respects, rather incomplete treatise so often referred to in this paper, I feel constrained to devote more space to its notice than I should otherwise have done.* "Probably no single dose," says Wood, "is capable, in the average man, of acting as a virulent

* Sir Thomas Watson says he has seen the good effect of the sedative doses, but he has apparently never used them.

poison, owing to but a limited portion of it being absorbed, the remainder being swept out of the alimentary canal by the diarrhœa produced." If Dr. Wood would experiment with this, as he has so carefully done with so many other not so valuable drugs, and uninfluenced by tradition and prejudice, he would find that it neither passes out of the bowels quietly, "like so much chalk," as Headland says, nor is swept out by diarrhœa; for, when a *genuine sedative dose* is given to a sick person, for it is no test to give it to a healthy animal, as the Edinburgh Committee did, say not less than xx to xxx grains, it will, as I have shown by numerous published cases (op. cit.), sometimes produce constipation, not unfrequently a complete arrest of peristaltic action in dysentery and cholera for twelve hours; and even when it acts as a cathartic it does so not very promptly, and generally in the mildest manner; and ptyalism is almost never induced. It is true that if an overdose is given it does pass quietly out of the bowels without injury, as Headland says, but he is in error when he says that only the *ordinary* cathartic dose takes effect, say 5 or 10 grains. For if in the cases above alluded to such a dose be given, it will usually, probably always, produce irritative and aggravating effects, whereas the full dose acts in a manner precisely the reverse. There is, probably, as Wood states, no such thing as a poisonous dose of the drug, but I have never had occasion to go further than 90 grains in one night to a child with membranous croup, and I have always seen either good effects or no effect at all from such quantity. I refer to an interesting case of Hamilton's, in the person of a child of the celebrated Sidney Smith, published in his biography. The country practitioner, frightened at the doses recommended by Hamilton, abandoned the case, and the father carried out the treatment and saved his son.

That calomel has its sedative influence by direct action on the peripheral nerves, and so on the nerve-centres, and that, when more is administered than is required for this effect, the surplus is not absorbed, and does not affect the blood, is not only sufficiently proved, as far as clinical evidence can prove it, by the large number of recorded cases of my own and of many other observers in this city, but especially in the Western and South-western States, but has remarkable corroborative evidence in the similar action of a remedy taken from the *vegetable* kingdom. Although one drop of croton-oil is a dose, and two or three, which are very rarely given, will often produce alarming diarrhœa and prostration, a drachm or more has been accidentally swallowed without serious results. "Cowan," says Wood (op. cit.), "has recorded the case of a child, four years old, who recovered in two days, from a teaspoonful of croton-oil taken on a full stomach." Adams, another case of recovery from a drachm taken by injection. Wood himself has only been able to discover two fatal cases from large doses. In one, the dose was two and a-half drachms, and the patient a man already worn out by a four weeks' illness from typhoid fever. In the other, recorded by Giacomini (Stille's Therap.), "twenty-four grains" proved fatal, not by purgation, there having been only four passages, but apparently by nervous shock, the symptoms being those of "general collapse, the patient preserving consciousness to the last." When injected into the veins, the weight of evidence from experimenters on animals is to the effect that it does *not* thus produce its characteristic effects (Hertwig and Bucheim). In certain conditions of the peripheral nerves of the intestines, as where they are poisoned by lead (Colica Pictonum), and obstinate constipation results, the ordinary dose of the oil has no effect. I have repeated the dose, one or two drops, every hour, until ten or fifteen drops were taken, without eliciting any response, and with no evidence of absorption into the blood. This peculiar action of lead on the intestinal nerves, renders its preparations a valuable remedy in some forms of diarrhœa, but the doses must be large enough, and they are seldom so, to produce this sedative effect. The fatal dose, in the latest work on Mat. Med., is one to two ounces (H. Wood). The danger of affecting the system seriously, is in giving small doses continuously—the almost infinitesimal doses which painters, plumbers, etc., sometimes get, producing all the serious phenomena of chronic lead-poisoning. So it is with calomel; there is absolutely no danger from a teaspoonful, while a few grains, given in minute doses, may produce the most serious consequences—in the one case, the medicine acting through the *nerves*, in the other, through the blood. This peculiar immunity from large doses of drugs, while small or moderate doses produce such serious consequences, deserves more investigation than it has received. Take *arsenic* for instance, reputed one of our most active poisons: while unpleasant consequences arise from fractions of a grain, and death, it is said, from two or three grains, there are instances on record where several drachms, in one case two ounces, were taken, and the patients recovered ; in one case a very large quantity was taken in lumps, and sixteen hours elapsed before remedies were applied. It has been said that these large doses cause prompt emesis, and thus a large portion is rejected ; and, in such cases as the last, that it was in a very insoluble form. But I have, within a few days, seen a remarkable case where none of these theories could be applied. I was invited by Dr. D. I. Bruner, of Columbia, Pa., to see the patient, and from the two I obtained a complete history.

Mr. W. B., aged 55, a healthy man, but rather addicted to the favorite German drink of that region, had been meditating suicide, as he informed me, for three weeks ; when, on the 19th Sept., 1874, having warmed up his courage by some five or six glasses of lager, he obtained an ounce of arsenious acid, in powder, from the principal druggist, under a specious pretext, and took full three-fourths of it, six drachms. He had just before taken some food. Supposing that death would come soon, he laid himself down in a proper position, informed his family of what he had done, and bade them good-by. His wife summoned Dr. B. at once. The doctor found him comfortable, but endeavored to persuade him to take an emetic, but he positively refused to have anything done. The doctor remained with him an hour and a half, and then left, supposing there must be some deception ; an hour after, however, he was again hurriedly called, and found the man in great agony, vomiting, purging, and complaining bitterly of the usual pains and burning sensations in stomach and bowels. He had taken the ipecac left by Dr. B. It was not until *five hours* after the ingestion of the poison that Dr. B. got the usual antidote, which he gave him in large quantity as fast as he rejected it. None of the arsenic was observed in the vomited matters ; but the doctor did not see the first. He gradually recovered from the pains, and fourteen hours after the dose was taken he fell into a quiet sleep, with a natural pulse, easy respiration, normal temperature (by the thermometer), and rested four hours, and awoke free from pain or burning, and has had none since. In fact, with the exception of the first few hours, he has had not one of the many characteristic symptoms of acute arsenic poisoning, and a hypodermic injection of morphine would, in all probability, have promptly relieved those from which he did suffer. The nausea and vomiting continued about four days, but during that time he took and relished all the food he was allowed to have, and his appetite and digestion have been unusually good up to the present time, seven or eight weeks. I said there were no characteristic phenomena of acute poisoning. He did have marked injection of the conjunctivæ, and a slight feeling of constriction of the pharynx for a few days. On the seventh day, symptoms of chronic poisoning set in, with numbness and pricking, closely followed by weakness of the feet first, then the hands, and in the course of a week increased to the degree in which they exist now. Nov. 12th, he can close his fingers on my hand, but cannot grasp. He cannot stir foot or toes ; can use the muscles of the thigh. The feet are very slightly œdematous, but only from letting them hang down most of the day. The functions of all his viscera appear to be perfect ; his tongue is clean. His pulse, sitting up, is 120, and rather feeble. Recumbent, pulse 90. He can feel pricking or pinching in almost all parts of the foot, and refer it to its proper locality, but the sensation is always that of burning. Has been on the iodide of potassium. Within the past week the doctor thinks he has improved a little. Recommended increase of the dose (10 grs.) of the iodide, strychnia hypodermically, and electricity.

Bromide of potassium sometimes acts in a similar manner as regards dose; some physicians have given it in half-ounce doses, with good results. One physician knew of a case in which an ounce was taken at a dose with no unpleasant result ; and a medical acquaintance, whose name I cannot now recall, had a patient, during the war, to whom an orderly gave a saturated solution, containing *two ounces*, for dispensary use, without its being known until the following morning. The effect was not greater than it sometimes is from half a drachm. A lady patient of mine took nearly half an ounce, in a state of desperation from insomnia, and showed no other bad effect than want of co-ordination of the muscles for some days. She was rather benefited otherwise. One unpleasant effect, however, of a concentrated solution, and of even the little lenticulars of Dunton, on an empty stomach, is a most violent though transient pain in the epigastrium.

The above phenomena have also been noted in the case of *quinine*. A case has recently been reported to me by Dr. Woodhull, a former surgeon of volunteers, where one of his men took an ounce of quinine, and no marked symptoms resulted. He sometimes gave half-ounce doses, and frequently doses amounting to half an ounce daily.* H. Wood, however, refers to the case of a man and wife, the former of whom took two ounces, and the latter one ounce, at one dose. The man died, and the wife "was recovered with difficulty." Many physicians believe that, after reaching a certain dose of the drug, say fifteen to thirty grains, you cease to get any response, and the larger doses are simply waste of a valuable drug. I am myself inclined to take this view, as in the case of calomel, half a drachm of which, at one dose, is as far, probably, as it is ever necessary to go.

(To be continued.)

* New York Med. Journal, Art. "On Hypodermic Treatment of Fever," by the Author.

